# Hemangiosarcoma in a Dog: Unusual Presentation and Increased Survival Using a Complementary/Holistic Approach Combined with Metronomic Chemotherapy

**DOI:** 10.1155/2018/6160980

**Published:** 2018-02-05

**Authors:** Philip Chaikin, Anja Welihozkiy

**Affiliations:** ^1^Chaikin Associates LLC, 8388 South Tamiami Trail, Sarasota, FL 34238, USA; ^2^Department of Pharmacy Practice and Administration, Ernest Mario School of Pharmacy, Rutgers University, New Brunswick, NJ, USA; ^3^Blue Pearl Veterinary Partners, 7414 South Tamiami Trail, Sarasota, FL 34231, USA

## Abstract

This case report documents the clinical and pathologic findings in a 12-year-old terrier mix with intraocular and splenic hemangiosarcoma. Pathologic findings in both the spleen and globe were consistent with hemangiosarcoma with a low mitotic count. Initial treatment consisted of enucleation and then splenectomy followed by one cycle of conventional doxorubicin chemotherapy. Due to poor tolerance, a subsequent treatment regimen consisted of metronomic chemotherapy with chlorambucil combined with an alternative/complementary regimen of I'm-Yunity (polysaccharopeptide) and Yunnan Baiyao. Follow-up thoracic radiographs and abdominal ultrasounds over a period of 24 months showed no evidence of pulmonary, hepatic, or right atrial metastases, during which time the patient had an excellent quality of life. However, shortly after achieving two-year survival, the patient developed new onset seizures unresponsive to anticonvulsant therapy. Therefore, a decision was made to euthanize the dog given that the most likely etiology of the seizures was a brain tumor. Overall, this is an exceptional treatment response given the poor survival statistics of hemangiosarcoma even with conventional chemotherapy. However, additional clinical pharmacology and clinical trial data are needed to further support the use of a complementary/holistic approach in combination with metronomic chemotherapy.

## 1. Introduction

Hemangiosarcoma is a very aggressive cancer in dogs with high mortality rate and median survival of 3–6 months with a ten percent one-year survival rate [1]. The majority of primary tumors are found in the spleen and many dogs will succumb to organ rupture and hemoabdomen. Following splenectomy, adjuvant doxorubicin based chemotherapy is usually offered to patients; however, it adds only several months of incremental survival [1]. Consequently, some owners decide not to subject their companion pets to aggressive intravenous chemotherapy given the toxicity profile. This has prompted dog owners to seek alternative therapies. In the past several years, there has been increased interest in metronomic low dose oral chemotherapy regimens as well as complementary/alternative holistic treatments. Metronomic chemotherapy usually consists of low dose oral cyclophosphamide or chlorambucil plus a nonsteroidal anti-inflammatory drug (NSAID). It is reasonably well tolerated with survival statistics similar to traditional intravenous cytotoxic chemotherapy [2].

Here, we describe a case report where hemangiosarcoma presented in an unusual manner with initial symptoms being ocular in nature. We also describe the pharmacology of metronomic chemotherapy in combination with a complementary/holistic approach of polysaccharopeptide (PSP) extract from the Coriolus versicolor mushroom (I'm-Yunity) and Yunnan Baiyao, two ancient Chinese medicines that have been evaluated in human cancer patients in the Chinese/Japanese medical literature. A pilot study conducted in fifteen dogs with metastatic hemangiosarcoma demonstrated encouraging results with PSP showing similar median survival times to standard doxorubicin chemotherapy (historical control data) [3]. This prompted the initiation of an ongoing pivotal clinical trial in 100 dogs with hemangiosarcoma treated with either PSP versus standard doxorubicin based chemotherapy versus the combination of chemotherapy plus PSP (personal communication with University of Pennsylvania School of Veterinary Medicine). It is important for clinicians to recognize and appreciate the pharmacologic potential of alternative treatment regimens given that some dog owners decide against treatment with intravenous adjuvant chemotherapy.

## 2. Case Description

A 12-year-old, 12 kg, spayed, female terrier mix was noted by her owner to have excess tearing and a collection of blood in her left eye. The primary care veterinarian diagnosed anterior uveitis and hyphema (OS). Serum chemistry revealed a mildly elevated AST of 73 (15-66) and CPK of 1267 (59-895). Complete Blood Count (CBC) revealed 6 nucleated RBCs/100 WBCs, and T4 was normal. Her primary care veterinarian attributed the hyphema to trauma and prescribed oral carprofen tablets (25 mg BID) and NeoPolyDex (neomycin, polymyxin B, and dexamethasone 0.1%) ophthalmic suspension (TID). After several weeks without improvement, the owner sought consultation with a veterinary ophthalmologist. Examination revealed continued anterior uveitis and development of iris bombe in OS, as well as early immature cataract in the right eye (OD). The OS showed a fixed pupil with absent light reflexes due to posterior synechiation. There was a normal palpebral reflex but absent menace response and very diminished, but present dazzle reflex. The present hyphema in OS prevented visualization of the posterior segment. The OD showed trace flare consistent with anterior uveitis. OD also showed normal pupillary light reflexes as well as normal palpebral reflex, menace response, and dazzle reflex. Tonometry revealed 14 mmHg in both eyes. The presence of bilateral uveitis was indicative of a systemic condition. Doppler oscillometry during the first visit revealed a systemic blood pressure of 85 mmHg which ruled out hypertensive retinopathy. An infectious disease panel was obtained and submitted. Dorzolamide 2% ophthalmic solution (OS TID) and atropine 1% ophthalmic ointment (OD for 3 days) were added to the treatment regimen. Follow-up evaluation showed the hyphema in OS to be resolved as was the uveitis in OD with the above treatment. In addition, the intraocular pressures were 9 mmHg OD and 15 mmHg OS. The left eye continued to show iris bombe; therefore, an ocular ultrasound was performed which revealed a retinal detachment and a preretinal hyperechogenicity suspected to be vitreous hemorrhage, but no evidence of an intraocular tumor.

Infectious disease fungal serology was negative for histoplasma, blastomyces, aspergillus, cryptococcus, and coccidiomycosis. Urine blastomyces antigen was negative.* Ehrlichia canis* titer was negative; Rocky Mountain spotted fever titer was 1 : 64 (normal < 1 : 64) which was suspected to be secondary to prior exposure as opposed to active infection. Toxoplasmosis ELISA IgM titer was negative but IgG titer was 1 : 256. CBC and chemistries were normal at this time with normal AST and CPK and no nucleated red blood cells. Clindamycin (150 mg po BID; 25 mg/kg/day) was prescribed for 30 days on an empirical basis to treat presumptive toxoplasmosis. A decision was eventually made to enucleate the OS as the eye was no longer visual (previously only demonstrating light perception), and the intraocular pressure had increased to 36 mmHg. Uveitis in the OD also became progressively worse necessitating topical treatment with NeoPolyDex ophthalmic suspension (TID), several topical nonsteroidal anti-inflammatories (diclofenac 0.1% ophthalmic solution, then flurbiprofen 0.03% ophthalmic solution, both TID), and atropine 1.0% ophthalmic ointment once weekly.

**Figure 1:**
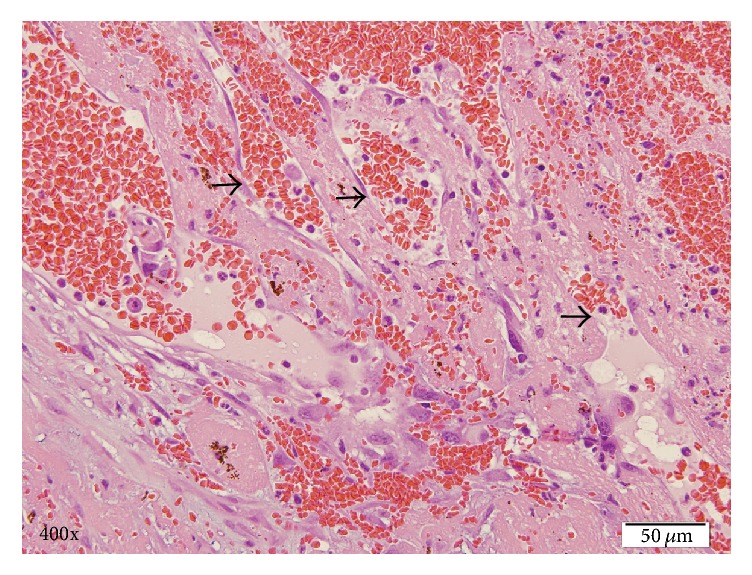
Hematoxylin and eosin stain of intraocular hemangiosarcoma demonstrating neoplastic spindle cells lining blood-filled vascular channels. Arrows indicate neoplastic cells lining vascular, blood-filled channels.

**Figure 2:**
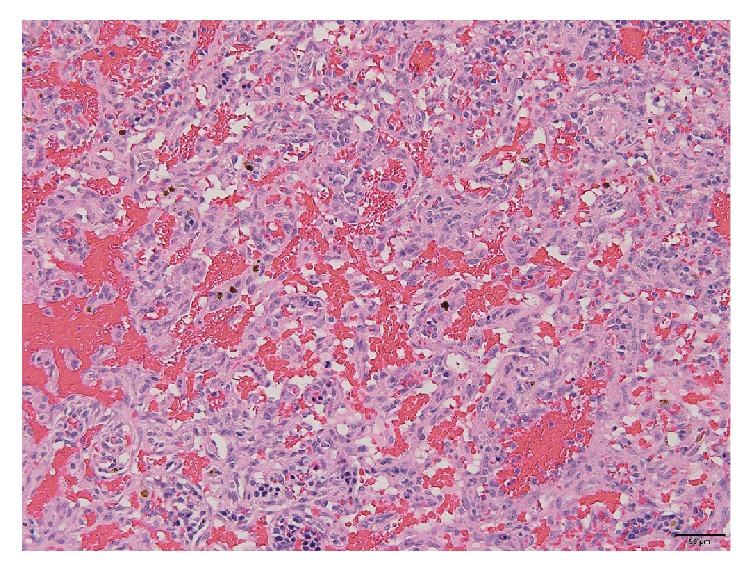
Hematoxylin and eosin stain of splenic hemangiosarcoma. Neoplasm consists of erratic streams of neoplastic, endothelial-like, fusiform cells forming the linings of tortuous blood-filled channels and cavities supported by fibrous stroma.

**Figure 3:**
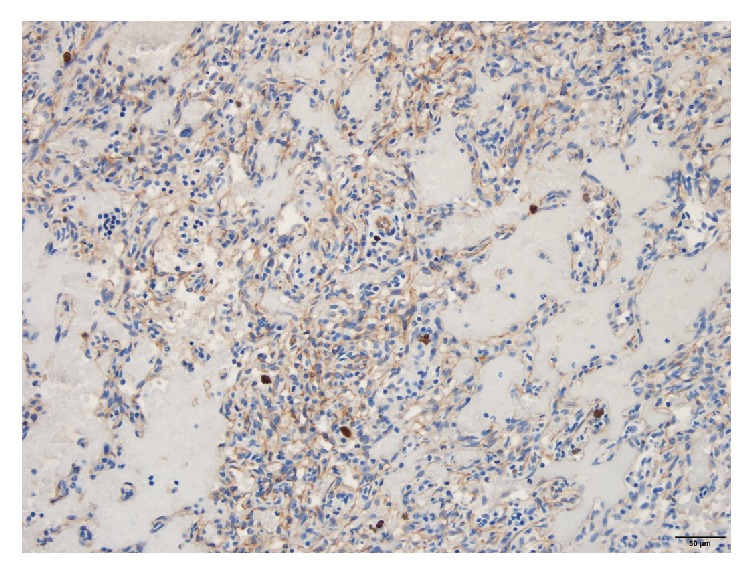
Positive immunohistochemical staining of the splenic tumor for CD31. Brown color indicates positive staining of neoplastic cells immunoreactive with a monoclonal mouse anti-human CD31 antibody.

The OS was submitted for histopathologic evaluation and revealed the following findings: (1) mesenchymal malignant neoplasia suggestive of metastatic hemangiosarcoma, (2) severe hyphema and hemorrhage in the posterior chamber and vitreous, (3) iatrogenic lens capsule rupture, and (4) chronic glaucoma. The presence of atypical fusiform cells, carpeting the posterior aspect of the iris and ciliary body surface, dissecting the hemorrhage in the posterior chamber, and forming vascular channels, was deemed to be suggestive of metastatic hemangiosarcoma to the eye (Figure 1). Pleomorphism was moderate with a few karyomegalic cells and mitotic figures. There was no significant inflammatory component that would be consistent with infectious uveitis. This diagnosis prompted a search for the primary neoplasm and further diagnostic work-up including thoracic radiographs and abdominal ultrasound. The ultrasound demonstrated an ill-defined, heterogeneous, partially cavitated mass measuring 3.4 cm in the spleen, distorting the normal contour. Thoracic radiographs were unremarkable. The patient underwent splenectomy and liver biopsy which showed a mass in the spleen measuring about 5 cm in diameter, still contained within the capsule. Intraoperative evaluation/inspection of the liver did not reveal gross abnormalities. There was no evidence of gross metastasis in the omentum or mesentery. The pathology report noted part of the splenic parenchyma to be effaced by a nonencapsulated, invasive, neoplastic growth with extensive tumoral necrosis and hemorrhage. The growth comprised erratic streams of neoplastic, endothelial-like, fusiform cells forming the linings of tortuous blood-filled channels and cavities supported by fibrous stroma (Figure 2). There was mild anisocytosis, mild anisokaryosis, and a low mitotic count (1 mitotic figure per 10 consecutive high power fields, 400x). To further confirm the diagnosis of splenic hemangiosarcoma, immunohistochemical analysis using a monoclonal mouse anti-human CD 31 antibody (DakoCytomation, Denmark) was conducted on the splenic tumor tissue using an automated slide staining system (IntelliPATH, Biocare Medical, Pacheco, CA). Presence of CD 31 (platelet and endothelial cell adhesion molecule) staining confirmed the tumor to be of vascular origin. As can be seen in Figure 3, the neoplastic cells were immunoreactive for CD31 antibody. The remaining splenic parenchyma was congested with extramedullary hematopoiesis. The liver biopsy showed hepatocellular hydropic degeneration.

A decision was taken by the owner to use conventional adjuvant chemotherapy with doxorubicin monotherapy. Following the first cycle at a dose of 28 mg/m2, the dog experienced several episodes of vomiting. No further cycles of intravenous chemotherapy were administered. After consultation with several schools of veterinary medicine and a veterinary oncologist, the owner decided on a regimen of I'm-Yunity (polysaccharopeptide from the Coriolus versicolor mushroom, dose of 100 mg/kg/day po), metronomic chlorambucil 1.0 mg po QD (2.0 mg/m2), high doses (900 mg) po QD of omega-3 fatty acids, and Yunnan Baiyao 250 mg po BID for two weeks on, one week off. Initially, firocoxib 57 mg po QD was used in combination with chlorambucil but was discontinued after two weeks due to gastrointestinal toxicity. Over the next 24 months, the patient maintained a stable weight, demonstrating high energy level and excellent quality of life. There were no adverse effects of the treatment regimen and the patient was on this combination continuously for two years. Her vision continued to deteriorate in the right eye due to progressive cataract formation and continued uveitis despite the use of flurbiprofen, tacrolimus, and NeoPolyDex TID. Serial thoracic radiographs and abdominal ultrasound obtained every 3-4 months over 24 months showed no evidence of visible pulmonary, hepatic, or right atrial metastases. Over the two-year time frame, the patient developed polyuria and polydipsia, mild anemia of chronic disease (Hgb/Hct = 13/34 decreased from 16/50), progressive increase in alkaline phosphatase (from 221 to 727, normal range 20–150), and rising BUN/creatinine (from 20/0.8 to 47/1.5 mg/dl) (IRIS stage 2 renal disease). Otherwise, her laboratory parameters were normal. Several days after achieving 24-month survival, the patient developed new onset tonic seizures. Phenobarbital was started at a dose of 16 mg po BID, increasing to 32 mg po BID. However, seizure frequency continued to increase with one to two episodes daily over the course of the next 10 days. Therefore, a decision was made to euthanize the patient given that the most likely cause of the seizures was a brain tumor.

## 3. Discussion

Hemangiosarcoma is an aggressive cancer and usually metastasizes to the lungs, liver, brain, and right atrium [4]. Although it is uncommon for the presenting symptoms to be ocular in nature, hemangiosarcoma is the most common metastatic tumor to the eye in dogs [5]. It is fortuitous that the cancer was initially diagnosed from the patient's ocular pathology which prompted identification of the primary tumor in her spleen. This allowed subsequent splenectomy prior to potential organ rupture and hemoabdomen leading to hypotensive shock, the most frequent cause of death from hemangiosarcoma.

Research over the last few decades has increased our understanding of the molecular pathways of cancer metastasis and drug resistance which has led to more targeted therapeutic agents and the use of continuous or uninterrupted low dose chemotherapy (metronomic chemotherapy). This treatment approach has emerged as an alternative to conventional cytotoxic chemotherapy which is based on using the identified maximal tolerated dose. The targets for metronomic chemotherapy include the tumor vasculature via an antiangiogenic and antiproliferative effect on endothelial cell precursors as well as an effect on certain immune cells that facilitate the tumor's ability to evade recognition and attack from the immune system [6]. Treatment responses to metronomic chemotherapy are therefore based on achieving durable stable disease rather than decrease in tumor volume. Many preclinical studies have confirmed the antiangiogenic effect of metronomic chemotherapy (inhibition of endothelial cell proliferation and migration, decreased mobilization of circulating endothelial progenitor cells, and induction of thrombospondin-1 and other endogenous angiogenic inhibitors) [6]. Several studies have also confirmed enhanced efficacy of metronomic chemotherapy when combined with an NSAID [7].

In addition, there is data to support that metronomic dosing of cyclophosphamide (an alkylating agent) is associated with multiple immune-stimulatory effects including decreases in the number and function of regulatory T cells, dendritic cell activation, and stimulation of cytotoxic T cells [7]. Tumors express a range of antigens, including self-antigens. Regulatory T cells are crucial for maintaining T-cell tolerance to self-antigens and are thought to dampen cell immunity to tumor associated antigens and to be the main obstacle tempering successful immunotherapy. Other studies have shown that metronomic cyclophosphamide or chlorambucil led to a decrease in the number of T regulatory cells in peripheral blood in dogs with soft tissue sarcomas [2, 6]. The goal of metronomic chemotherapy is to achieve stable disease with acceptable quality of life. By targeting essential tumor associated survival pathways such as angiogenesis and immune escape mechanisms, metronomic chemotherapy may be more appropriate and less toxic than conventional intravenous chemotherapy [6].

Coriolus versicolor (CV) is a medicinal mushroom known as Yun Zhi in China. The chemical composition of CV is complex with several classes of compounds suggested to be responsible for its biologic activities. However, polysaccharopeptide (PSP) is considered to be a major active moiety. PSP represents a homogenous mixture of macromolecules with closely similar physicochemical characteristics; therefore, isolation of a single pure PSP for structural elucidation is technically difficult [8]. Chemical techniques have suggested that PSP is a group of polysaccharides chemically linked to various peptides [8]. Coriolus versicolor mushroom contains two polysaccharopeptides: PSP and PSK derived from the Cov-1 strain and CM-101 strains, respectively. Both demonstrate in vitro and in vivo immune-restorative and antitumor activities [8]. The PSP product used in the University of Pennsylvania pilot study and to treat this patient was I'm-Yunity (Integrated Chinese Medicine Holdings Ltd., Hong Kong), with each capsule containing 400 mg of PSP, manufactured according to GMP standards by Formulation Technology Inc., USA [3]. PSP in the form of I'm-Yunity is isolated from the mycelium of the mushroom, not the fruiting body as is the case with other brands [3]. Quality control procedures are in place for I'm-Yunity according to GMP standards.

PSP has been shown to demonstrate interesting pharmacologic properties in vitro with activation of T lymphocytes, B lymphocytes, natural killer cells, and lymphocyte-activated killer cells in addition to promoting the proliferation and/or production of antibodies and various cytokines such as interleukin 2, interleukin 6, interferons, and tumor necrosis factor [8]. I'm-Yunity has demonstrated antitumor activities in tissue culture studies causing cell cycle arrest and alterations in the expression of apoptogenic/antiapoptotic and extracellular signaling proteins with a net result being a decrease in tumor proliferation and an increase in apoptosis [9]. In vitro studies have demonstrated I'm-Yunity to cause a dose and time dependent growth suppression and decreased cell viability for HL-60 leukemic cells [3]. Other in vitro studies have demonstrated cytotoxic activity against several tumor cell lines for gastric and lung cancer, leukemia, and lymphoma [8]. PSP has also demonstrated antitumor activity in vivo with significant reduction in tumor size in mice inoculated with various cancer cell lines (leukemia, nasopharyngeal, liver, sarcoma, hepatoma, melanoma, and colon) [3, 8]. The use of CV extracts has been evaluated in humans as a therapeutic adjuvant for cancer immunotherapy in a variety of solid tumors in numerous human clinical trials in China and Japan demonstrating an increase in immune cell proliferation, decrease in chemotherapy symptoms, enhancement of tumor infiltration by dendritic cells, and cytotoxic T lymphocytes as well as prolongation of relapse free periods and overall improvement of prognosis [3, 8].

The pilot study of I'm-Yunity conducted at University of Pennsylvania School of Veterinary Medicine demonstrated median survival times of 117 days and 199 days for 50 and 100 mg/kg doses of I'm-Yunity, respectively, in fifteen dogs with metastatic hemangiosarcoma [3]. These survival times are greater than those for splenectomy alone (median survival times ranging from 19 to 86 days) and comparable to doxorubicin based chemotherapy (reported median survival times of 141–179 days) [3]. However, it is important to note that currently there are no peer reviewed published randomized clinical trial data comparing I'm-Yunity to doxorubicin. The pivotal trial that will provide answers to these questions is still ongoing (personal communication with University of Pennsylvania School of Veterinary Medicine).

CV extracts (PSP) are very well tolerated by dogs and humans with minimal adverse events. The reported LD_50_ of Coriolus extracts given to mice is greater than 18 g/kg and there were no significant abnormalities reported in acute and chronic animal toxicology studies [8]. Doses of up to 15 grams per day of PSP have been tolerated long term in humans without noticeable adverse events [10]. Due to immune-stimulatory effects of CV extracts, there is the potential for decreased activity of immunosuppressant drugs and this combination should be avoided. There are no reported pharmacokinetic drug interactions for I'm-Yunity with cytochrome P450 3A4 substrates [11].

Yunnan Baiyao is another Chinese herbal medicine that has been utilized for its hemostatic and wound healing properties in people. It is frequently used in veterinary medicine to control bleeding in dogs with hemangiosarcoma by improving clotting and platelet function [12]. Yunnan Baiyao is a protected Chinese traditional medicine and the exact herbal formula is a trade secret. However, global demand for quality assurance and manufacturing according to GMP standards has allowed the product to be labelled in order to identify its major components. A major component is Panax notoginseng root extract which has demonstrated growth inhibition and increased apoptosis of SW 480 human colorectal cancer cells in vitro [12]. It has also been shown to inhibit DNA synthesis and cell proliferation in human umbilical vein endothelial cells in vitro [12]. The wild yam root component of Yunnan Baiyao has been shown to cause apoptosis of a murine neuroblastoma cell line and also to induce antiproliferative and proapoptotic effects in a range of tumor cells by G2/M arrest, downregulation of NF-kB, and other pathways [12]. There is a body of literature showing that the various components of Yunan Baiyao have anticancer properties which prompted a recent study conducted at the University of Florida School of Veterinary Medicine. This study evaluated Yunnan Baiyao against three canine hemangiosarcoma cell lines in vitro and demonstrated a time and concentration dependent cell death due to caspase induced apoptosis in all three cell lines [12]. The average in vitro IC50 concentrations for cell death are believed to be similar to the in vivo mean steady state plasma concentrations achieved with multiple dosing of Yunnan Baiyao [12]. However, additional clinical data are needed for a more thorough understanding of the pharmacokinetics and pharmacodynamics of Yunan Baiyao and its major active ingredient (Panax notoginseng). Holistic veterinary practitioners use a regimen of 250 mg BID for two weeks on and one week off or two months on and one month off. There is a need for additional clinical trial data to confirm the dosing regimens of Yunnan Baiyao currently used. Vacation periods are offered due to the potential for elevation in liver enzymes with continuous dosing. However, other practitioners use continuous daily dosing of Yunnan Baiyao (250 mg BID) without interruption (personal communication with Dr. Jason Schmalberg, Division of Integrative Medicine, University of Florida School of Veterinary Medicine). It seems that there is significant potential for additive or synergistic pharmacologic properties of I'm-Yunity and Yunnan Baiyao to be used together with metronomic chemotherapy in patients with metastatic hemangiosarcoma; however, additional clinical trial data are needed.

Given the prolonged survival of this patient, an important question to pose is whether this is a case of metastatic disease to the eye or two simultaneous, primary tumors. A low mitotic count was noted in both the splenic and ocular pathology reports compatible with a low doubling time of the tumor. One could suggest that the history of a splenic mass and the presence of atypical mesenchymal neoplastic cells forming vascular channels within the posterior chamber of the eye and choroid are suggestive of metastatic (likely splenic) hemangiosarcoma to the eye. As stated previously, hemangiosarcomas are the most common metastatic tumor to the eye in dogs [5]. Although the low mitotic count noted on pathology might be indicative of a less proliferative tumor with lesser propensity for early metastasis, it may not be reflective of the entire tumor tissue due to areas of extensive necrosis, which would actually be associated with more aggressive growth. This could be one possible explanation for the low mitotic count in the splenic tumor despite presumed early metastasis to the eye. An alternative hypothesis for this patient's prolonged survival and absence of additional tumor metastases after two years, and one that is perhaps more plausible, is that in many, if not all cases, hemangiosarcoma cells circulate early after the malignant transformation event and colonize various tissues and organs where tumors can develop independently. So it would not be surprising if this patient had two simultaneous primary tumors in her eye and spleen (personal communication with Dr. Jaime Modiano, University of Minnesota College of Veterinary Medicine, and Dr. Shi-Hsuan Hsiao, University of Illinois College of Veterinary Medicine). Unfortunately, there are no definitive techniques available to confirm one hypothesis versus the other.

A brain tumor was the most likely etiology of the new onset seizures which occurred two years after initial diagnosis of hemangiosarcoma. A necropsy was not performed; therefore, it is unknown whether this represented an intracranial hemangiosarcoma or a primary brain tumor unrelated to the underlying cancer. If this were an intracranial hemangiosarcoma, it is possible that the brain represented a sanctuary site unresponsive to the treatment regimen. It is also unknown whether I'm-Yunity and Yunnan Baiyao are able to cross the blood brain barrier.

New advances are being made towards a better understanding of hemangiosarcoma tumor genomics and immunotherapy/tumor vaccines [13]. It is imperative that funding be made available for a more thorough understanding of the tumor genomics of this devastating canine cancer so that better treatment modalities can be developed.

Along these lines in human medicine, the National Cancer Institute is currently sponsoring the* Exceptional Responders Initiative* which is analyzing tumors from exceptional responders hoping to identify the genetic and molecular changes that underlie individual patient responses to cancer treatment [14]. Tumor tissue RNA and DNA sequencing is conducted as well as an evaluation of the number of copies of certain DNA segments with the hope of evaluating the abnormal biology of cells that may have contributed to exceptional treatment response. One finding to date is that between 10 and 20 percent of various tumors from exceptional responders have unusually high numbers of genetic mutations which may make them more susceptible to immune attack [14]. There is also some evidence that immune cells may infiltrate tumors from exceptional responder cases more effectively than other tumors. These clinical data may also reveal biomarkers that could be used to predict treatment response to the same or similar treatment regimens in other patients. In the future, it is hopeful that these kinds of data may also have application to canine cancer treatment responses.

In conclusion, we present an unusual clinical presentation of hemangiosarcoma to the eye as the initial signs/symptoms coupled with a treatment regimen of metronomic chemotherapy and holistic/alternative treatments. This regimen resulted in an exceptional treatment response with a survival of two years with very good quality of life. More practitioners should be made aware of the utility of the combination of metronomic chemotherapy with I'm-Yunity and Yunnan Baiyao as an alternative to conventional doxorubicin based chemotherapy. Additional studies are needed to fully elucidate the clinical pharmacology profile of these agents.
